# A Simple Kit for the Good-Manufacturing-Practice Production of [^68^Ga]Ga-EDTA

**DOI:** 10.3390/molecules28166131

**Published:** 2023-08-18

**Authors:** Monika Skulska, Lise Falborg

**Affiliations:** Department of Nuclear Medicine, Gødstrup Hospital, 7400 Herning, Denmark

**Keywords:** [^68^Ga]Ga-EDTA, PET, renography, GMP-production, Kit, ^68^Ga-colloids

## Abstract

Glomerular filtration rates for individual kidneys can be measured semi-quantitatively by a gamma camera using [^99m^Tc]Tc-DTPA, with limited diagnostic accuracy. A more precise measurement can be performed on a PET/CT scanner using the radiotracer [^68^Ga]Ga-EDTA, which has been validated in animal studies. The purpose of this study was to develop an easy kit-based synthesis of [^68^Ga]Ga-EDTA that is compliant with good manufacturing practice (GMP) and applicable for human use. The production of the cold kit and its labeling were validated, as were the radiochemical purity measurement and analytical procedures for determining the Na_2_EDTA dihydrate content in the kits. In this study, we validated a GMP kit for the simple production of [^68^Ga]Ga-EDTA, with the intention of applicability for human use.

## 1. Introduction

With the current widespread distribution of positron emission tomography (PET) scanners, the interest in gallium-68 (^68^Ga)-labeled radiopharmaceuticals has increased. ^68^Ga’s high positron-emission fraction (89% maximum energy; 1899 keV) and its 67.71 min half-life provide sufficient levels of radioactivity for high-quality images, while minimizing the radiation dose that is given to patients [1]. The parent radionuclide, germanium-68 (^68^Ge), with a half-life of 271 days, provides an easily available method of producing ^68^Ga, with a shelf-life of approximately one year, from an efficient and medically approved ^68^Ge/^68^Ga generator [2].

Early ^68^Ge/^68^Ga-generators were eluted using ethylenediaminetetraacetic acid (EDTA) and provided the direct production of [^68^Ga]Ga-EDTA for use in brain imaging as well as the quantitative assessment of blood–brain barrier abnormalities that are associated with multiple sclerosis [3,4]. However, since the equilibrium constant for the formation of the complex is high (K_ML_ = 7.9 × 10^18^), the [^68^Ga]Ga-EDTA complex has high thermodynamic stability and, therefore, its decomposition is difficult [5]. This drastically limited the development of other ^68^Ga-labelled radiopharmaceuticals for these early radionuclide generators. Consequently, modern ^68^Ge/^68^Ga-generators use acidic eluent (hydrochloric acid) and provide ^68^Ga in cationic form to enable further labeling chemistry [5].

Therefore, ^68^Ga is also used to label ligands, such as peptides, antibodies, or hormones, which can be targeted to specific biologically accessible proteins, such as receptors, that are over-expressed by tumor cells. Examples include [^68^Ga]Ga-DOTATATE, [^68^Ga]Ga-DOTANOC, and [^68^Ga]Ga-DOTATOC, all of which play important roles in the diagnosis of neuroendocrine tumors due to their affinity with somatostatin receptors [6], or ^68^Ga-PSMA, which is used for the clinical imaging of prostate cancer [7].

[^68^Ga]Ga-EDTA is known to be cleared from the blood via the kidneys with a rate that depends on the renal glomerular filtration function [8]. In 2016, Hofman et al. proved that [^68^Ga]Ga-EDTA can be used as a substitute for [^99m^Tc]Tc-DTPA, which is used in conventional gamma camera single-photon nuclear medical imaging for a wide variety of clinical indications [9]. The quantitative capabilities of PET, combined with its inherent ability to perform 3D tomographic imaging, provide major advantages over conventional planar imaging, as has been shown in recent animal studies [10,11]. However, earlier methods for the production of [^68^Ga]Ga-EDTA, as, described in the literature, cannot be directly transferred to a GMP-compliant production method for human use. Therefore, we aimed to implement a local kit-based synthesis, analogous to the standard ^99^Mo/^99m^Tc-generator/kit preparation of radiopharmaceuticals used for gamma-camera and single-photon emission computed tomography (SPECT) examinations.

The goal of the present work was to develop, establish, and validate a kit-based production of [^68^Ga]Ga-EDTA, in which labeling takes place in a simple one-pot synthesis, where the generator is eluted into a vial containing disodium EDTA dihydrate. We developed a kit (hereinafter referred to as the EDTA kit) containing EDTA and the necessary buffer system. The production of an EDTA kit and the ^68^Ga-labeling reaction to obtain [^68^Ga]Ga-EDTA must be GMP-compliant and in accordance with the national regulations of the Danish Medicine Agency (DMA).

## 2. Results

### 2.1. EDTA Kits

#### 2.1.1. EDTA-Kit Composition

The composition of EDTA kits was designed with respect to the following requirements: appropriate amounts of reagents to provide the correctly labeled product without toxic effects; an appropriate ion strength and pH for intravenous injection and the correct pH to avoid possible side reactions during the labeling reaction. At pH values higher than 3, ^68^Ga^3+^-ions form oxide or hydroxide species of the ^68^Ga^3+^-ion with low solubility, some of which form insoluble colloids [12]. The introduction of buffers that act as stabilizing ligands in the reaction mixture prevents the formation of colloids and supports complexation with the intended ligand, which, in our case, is EDTA. Bauwens et al. showed that the optimal buffer choices for the radiosynthesis of ^68^Ga-Dotatoc are HEPES, acetate, or succinate with a pH of 3.5–5.0 [13]. The colloids are impurities, which are hereinafter referred to as ^68^Ga-colloids. The composition of a single EDTA kit is presented in Table 1. Such kits are stored in a freezer. The constraint requirements for kit design are further described in the Discussion section of this article.

#### 2.1.2. EDTA Kit Validation

A GMP-compliant production of three batches of the EDTA kit was performed. A comparison of the measured parameters with the pharmaceutical/chemical specifications showed that the production of the EDTA kit using the described method was robust and highly reproducible (Table 2). Stability studies of the EDTA kits were performed over a period of up to 14 months by executing repeated measurements of pH and full quality control (QC) programs for the labeling of the three validation batches.

#### 2.1.3. Na_2_EDTA Dihydrate Content Determination in EDTA Kits

**Figure 1:**
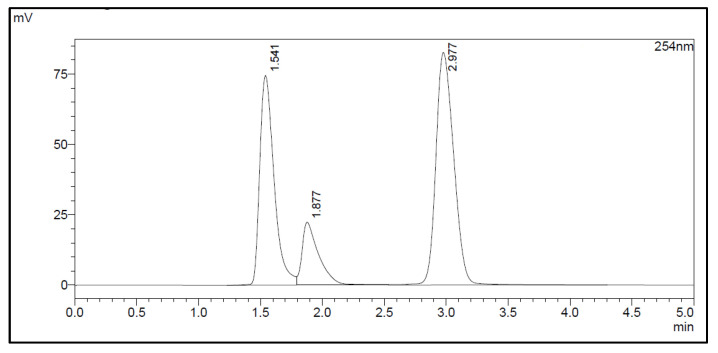
HPLC chromatogram.

**Figure 2:**
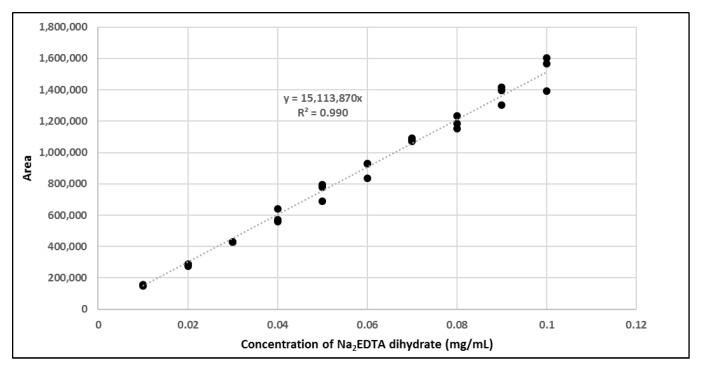
Calibration curve with area of Fe-EDTA peak as a function of concentration of Na_2_EDTA dihydrate.

The amount of Na_2_EDTA dihydrate (Table 2) in the EDTA kits was determined using a complexation reaction with Fe^3+^ followed by HPLC analysis, Figure 1. The peak at 1.5 min corresponds to Fe^3+^ ions, the peak at 1.9 min to Fe(OAc)_3_ and the peak at 3.0 min to Fe-EDTA [14,15,16]. A calibration curve was produced by analyzing solutions with various known concentrations of disodium EDTA dihydrate. The curve was constructed by plotting the area under the Fe-EDTA peak as a function of Na_2_EDTA dihydrate concentration (Figure 2). The linearity of the investigated EDTA concentrations was validated for the range 0.01–0.1 mg/mL. Samples of EDTA kits were diluted by a factor 10 prior to complexation with Fe^3+^, followed by HPLC analysis. Thus, using the slope of the standard curve, the concentrations of Na_2_EDTA dihydrate in the three batches of EDTA kit were 0.64, 0.69, and 0.69 mg/mL, respectively.

#### 2.1.4. Buffer Capacity: Labelling Process’ Robustness

The buffer capacity of the kit, both during and after elution of the generator, is important for the robustness of the overall labelling process. Thus, the challenge was to design an EDTA kit and labelling process that did not lead to stable insoluble ^68^Ga-colloid production as a radiochemical impurity whilst eluting the generator into the EDTA kit. Since the EDTA kit itself had a high pH value and the formation of ^68^Ga-colloid is known to take place at moderately acidic-to-basic conditions, the elution needs to be fast enough to obtain a low enough pH in time to avoid the formation of these impurities. The quality control results of the labelled product show that this was achieved.

**Figure 3:**
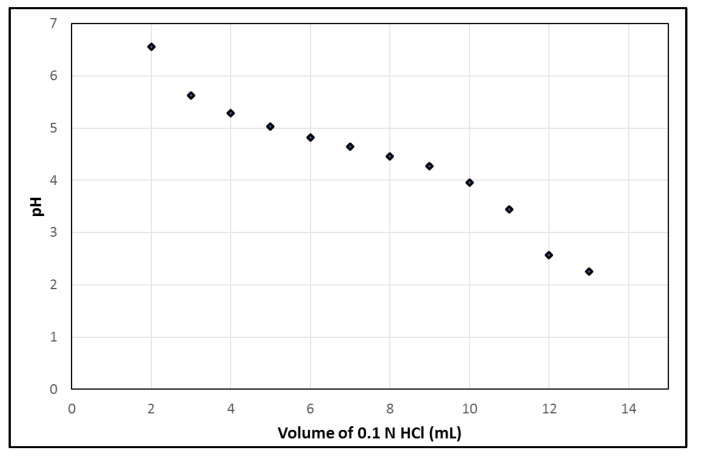
pH of EDTA kit during the addition of different volumes of 0.1 N HCl.

Additionally, using the syringe module of the PharmTracer, the generator is eluted with 7 mL 0.1 N HCl, 2 mL/min. However, small leaks in the cassette can cause a reduced volume of 0.1 N HCl. Therefore, the pH of the solution in the final product vial after complete elution of the generator should be stable and robust for varying volumes of 0.1 N HCl. Figure 3 illustrates the pH profile w.r.t. the addition of different volumes of 0.1 N HCl into an EDTA kit, which mimics the pH of [^68^Ga]Ga-EDTA with different volumes of 0.1 N HCl added from the generator during the elution of the generator into the vial. A pH of 4.65, which is optimal for a good labelling reaction as well as avoiding the formation of ^68^Ga-colloid and having an appropriate pH for i.v. injection, was obtained by the addition of 7 mL. The labelling process robustness showed a resultant pH range of 4.5–5.0 in the situation where the volume of eluent differed due to possible variations of ±2 mL in the automatic dispensing of the eluent while eluting the generator.

### 2.2. [^68^Ga]Ga-EDTA

Nine labelling procedures were performed using Modular-Lab PharmTracer: three for validation of the radiolabelled product, three for bioburden testing and three for stability studies. Three different batches of EDTA kits were used for each of the above procedures.

#### 2.2.1. EDTA Kit Labelling with ^68^Ga

^68^Ge/^68^Ga-generator qualification and validation were conducted prior to use. The identity of ^68^Ga was confirmed by radionuclide purity testing (half-life = 68.7 min; ^68^Ge-breakthrough = 0.00003% and gamma spectrum analysis (only 511 keV and 1077 keV photons characteristic to ^68^Ga were detected). The microbiological testing of eluate (sterility and endotoxin levels) detected no microbial contamination. The generator was eluted a minimum of 24 hours prior to labeling.

During synthesis, 7 mL of ^68^Ga-eluate was automatically transferred by the PharmTracer directly to the EDTA kit, where the conjugation reaction proceeded immediately. Bioburden testing showed no growth in the controlled batches. Specifications and results of the three validation runs are presented in Table 3.

The stability studies proved stability up to 2 hours after end of synthesis (EOS). The specification for bioburden was less than one colony-forming unit (CFU) per 10 mL (<1 CFU/10 mL) of the product. The test for bioburden in the three batches resulted in 0 CFU.

#### 2.2.2. Paper Chromatography of [^68^Ga]Ga-EDTA

Quality control of [^68^Ga]Ga-EDTA included the analytical procedures shown in Table 3.

**Figure 4:**
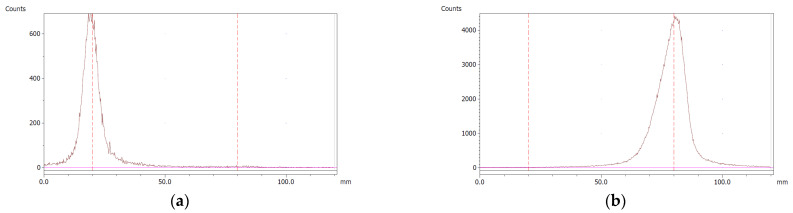
Chromatograms of (**a**) ^68^Ga-colloid; (**b**) [^68^Ga]Ga-EDTA.

The paper chromatography method for the determination of the radiochemical purity of [^68^Ga]Ga-EDTA was performed using Whatman Grade 1 Chr paper as the stationary phase and 0.9% sodium chloride as the mobile phase. The plate was 2 cm × 12 cm, and the sample was added 2 cm from the bottom edge and developed to 8 cm from the bottom. Typical chromatograms of ^68^Ga-colloid and [^68^Ga]Ga-EDTA are shown in Figure 4. The method is specific, precise and robust.

Validation, with respect to the specificity (successful) and accuracy (attempted) of the paper chromatography method for the determination of the radiochemical purity of [^68^Ga]Ga-EDTA, was performed according to EANM guidelines for the validation of analytical methods for radiopharmaceuticals [17].

To determine the method’s specificity, individual chromatograms were produced of ^68^Ga-colloid and [^68^Ga]Ga-EDTA. The resolution factor (Rs) was determined to be 3.70 from [17]:

$$\mathrm{Rs}=\frac{1{.18a(R}_{2}-R_{1})}{W_{h2}-W_{h1}}$$

where R_1,2_ are the retention factors, W_h(1,2)_ are the peak widths at half-height and α is the migration distance of the solvent front. Indices 1 and 2 stand for ^68^Ga-colloid and [^68^Ga]Ga-EDTA, respectively. The requirement for Rs is typically higher than 1.5.

**Figure 5:**
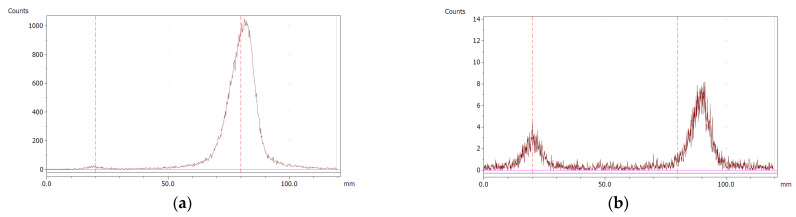
Chromatograms of (**a**) [^68^Ga]Ga-EDTA spiked with 3% of ^68^Ga-colloid.; (**b**) EDTA kit mixed with ^68^Ga-colloid. In both chromatograms, Region 1 corresponds to ^68^Ga-colloid and Region 2 corresponds to [^68^Ga]Ga-EDTA.

To evaluate the accuracy of the method, a standard solution of [^68^Ga]Ga-EDTA spiked with a known activity of ^68^Ga-colloid (3.0%) was analyzed to determine the amount of impurity in the product using the analytical method. Only 1.8% of activity of the ^68^Ga-colloid was detected (Figure 5a). By mixing an EDTA kit with ^68^Ga-colloid, [^68^Ga]Ga-EDTA was formed, indicating that ^68^Ga-colloid can be unstable in the presence of a strong chelator EDTA (Figure 5b). Due to this, it was not possible to design a method to determine the accuracy.

The analysis results were independent of whether the sample run was performed immediately after application of the spot or the spot was allowed to dry first. Thus, the method was robust. It was also precise, as shown from repeated measurements and comparison of the individual results (Table 3).

See the Discussion section for a further description of the development of the paper chromatography method used for this validation.

## 3. Discussion

Positron Emission Tomography has become a widespread diagnostic technique, which provides the possibility of a both accurate quantitative and qualitative assessment of physiological processes. ^68^Ga is one of the most common radionuclides used in PET-imaging. ^68^Ga conjugated with EDTA is a physiologically stable metal chelate that can be used for glomerular filtration rate (GFR) estimation and is reported to be suitable for renal function assessments. Gündel et al. have investigated and demonstrated the suitability of [^68^Ga]Ga-EDTA as a tracer for GFR calculation from PET-imaging in small animals, which is shown to conform well to the gold standard of inulin-based GFR-measurement. He also found that [^68^Ga]Ga-EDTA had no protein binding, whereas [^68^Ga]Ga-DTPA had a high level of protein binding, which resulted in the underestimation of GFR [18]. Others have also demonstrated the potential of this radiotracer for split GFR calculations in animals and expect it to have clinical application in human patients in the coming years [10,11].

At present, however, there is no commercially available cold kit for the preparation of [^68^Ga]Ga-EDTA that allows for diagnostic use directly after labeling, and hence has easy applications in clinical practice. The aim of this work was, therefore, to develop and validate a simple cold-kit, stored as a solution in a freezer, to enable the easy production of [^68^Ga]Ga-EDTA for clinical use, which conforms to the regulations set by the Danish regulatory authorities (DMA).

### 3.1. Determination of EDTA Kit Composition

Determination of the simple cold-kit composition provided a sterile and soluble product with the correct pH for both the conjugation reaction and the final labelled [^68^Ga]Ga-EDTA product. The volume of the labelled product was set to 10 mL and the volume of 0.1 N HCl eluent from the ^68^Ge/^68^Ga-generator was set at 7 mL. Consequently, the volume of the EDTA kit was 3 mL. Thereafter, the composition of the EDTA kit was designed with the following order.

#### 3.1.1. Amount of Na_2_EDTA Dihydrate

The concentration of Na_2_EDTA dihydrate in the radiolabelled product was determined to be 0.0005 M. This corresponded to 0.005 mmol in each EDTA kit and 0.0620 g in 100 mL EDTA kit solution. With a maximum injected volume of 10 mL, i.e., a maximum injected amount of Na_2_EDTA of 1.86 mg (which was also the total amount in an EDTA-kit), this corresponded to the EDTA kit containing 3 mL, with a concentration of Na_2_EDTA dihydrate of 0.62 mg/mL.

For higher concentrations, a better radiochemical yield of the complexation reaction is to be expected; however, as a trade-off, more toxicologic considerations need to be applied. Our aim was to maintain a low concentration of Na_2_EDTA dihydrate. Na_2_EDTA is used in therapy doses with a maximum of 3 g over 24 hours for the emergency treatment of hypercalcemia and the control of ventricular arrhythmias associated with digitalis toxicity [19]. Other concentrations of Na_2_EDTA have been reported in the literature, e.g., [^51^Cr]Cr-EDTA, which was previously used to measure GFR, with EDTA doses of up to 50 mg [20], and similarly for [^68^Ga]Ga-EDTA administered by i.v. injection, with doses of 0.05 M (18.6 mg/mL) with a maximum injected volume of 10 mL (i.e., a maximum injected Na_2_EDTA of 186 mg [9]). In this context, the 0.0005 M concentration of Na_2_EDTA dihydrate in our radiolabelled product was low.

#### 3.1.2. Amount of NaOAc Trihydrate

The amount of NaOAc trihydrate in the radiolabelled product was set to 0.1 M, as per Hofman et al. [9], corresponding to 1.00 mmol in each EDTA kit and 4.54 g in 100 mL EDTA kit solution.

#### 3.1.3. Amount of NaOH

The amount of 3 M NaOH was adjusted according to the following principles: half of the sodium acetate (0.5 mmol in each EDTA kit) should be protonated to offer a good acetate buffer capacity. This was achieved during the addition of 0.1 N HCl from the generator, where a total amount of 0.7 mmol HCl was added. The excess of 0.2 mmol of HCl should be neutralized by NaOH contained in the EDTA kit, resulting in 2.22 mL of 3 M NaOH in the 100 mL EDTA kit solution.

The kit was designed for use with a GalliaPharm ^68^Ge/^68^Ga-generator (Eckert&Ziegler) using 0.1 N HCl for elution. If other generators are considered, with other eluents or other volumes of eluent, the design can be adjusted accordingly, following the description and rationales provided above. However, this will require a separate validation.

### 3.2. Na_2_EDTA Dihydrate Concentration Determination as a Quality Control of EDTA Kit

The HPLC method used to determine the concentration of Na_2_EDTA dihydrate was implemented as a quality control test of the EDTA kit. Since EDTA itself does not absorb UV light, a Fe^3+^ complex was formed, which could be measured by UV detection on a HPLC system [14,15,16]. The method described in the following determined the concentration of Na_2_EDTA dihydrate with a certainty of ±10%, which was acceptable as we only required a rough estimate of the content to ensure no larger error was introduced during production. Furthermore, the amount given to the patient depends on the radioactivity concentration at the time of injection. This precision could be enhanced by introducing an internal standard in the chromatographic method; however, this was not the scope of this work.

### 3.3. Development of Labeling Method

An automatic, preprogrammed production method using the ModularLab PharmTracer was used to ensure the radiation safety of the personnel. The method needs to be rapid, reproducible and yield a high radiochemically pure product. Labeling reactions were carried out in the disposable cassettes, providing an easy and fast method that routinely achieved very high radiochemical yield and purity >99%. Elution of the generator into the kit takes 3.5 minutes and the complexation reaction between ^68^Ga^3+^ and EDTA takes place immediately. Thus, labeling of the product can be performed immediately prior to diagnostic examinations, ensuring minimum loss of radioactivity between production and patient administration.

### 3.4. Development of Chromatographic Method for [^68^Ga]Ga-EDTA

Thin-layer chromatography (TLC) and paper chromatography are commonly utilized, easy methods for the determination of impurities in radiopharmaceuticals. We aimed to develop a method to determine the formation of the ^68^Ga-colloid impurity in the [^68^Ga]Ga-EDTA product. Ga^3+^ ions are prone to hydrolysis in aqueous solutions and form different mono- and polynuclear hydroxide species depending on pH, temperature and ionic strength conditions [12]. Free ^68^Ga-ions are not expected in the product, since the strong chelator EDTA will ensure that all ^68^Ga-ions are coordinated to EDTA (details on experimental proof are explained at the end of this section). Technically, it should be possible to separate [^68^Ga]Ga-EDTA and ^68^Ga-colloid, so we investigated the chromatography systems described in the literature for ^68^Ga-labelled peptides and [^68^Ga]Ga-EDTA to determine their applicability to our system.

For this investigation, we wanted to prepare the ^68^Ga-colloid impurities. During our studies, the method for its preparation, as described in the Ph.Eur. [21], was replaced by the Bench titration method [22], which provides a more precise pH adjustment and which, in our opinion, is a superior method. The ^68^Ga-colloid formation was influenced by pH, and unstable oxides or hydroxides were able to complex with EDTA, leading to [^68^Ga]Ga-EDTA [23].

The applied methods using iTLC-SG as the stationary phase are summarized in Table 4. One examined method was based on the analysis of the radiochemical purity of [^68^Ga]Ga-PSMA-HBED-CC in the Ph.Eur. monograph [21], which provides a nice sharp peak at Rf = 0.0 for the ^68^Ga-colloid. However, this method was not suitable for [^68^Ga]Ga-EDTA, since this complex provided a broad tailing peak (Table 4, entry 1). The three other applied methods (Table 4, entries 2–4) had the common feature that the ^68^Ga-colloid peak did not stay at Rf = 0, which, according to the literature, was to be expected [24,25,26]. A plausible reason for this is that the ^68^Ga-colloids used as reference samples in ours and published studies may not have the same stability. In our studies, it could be argued that the eluents containing either EDTA or TFA can lead to transchelation from ^68^Ga-colloid, or rather ^68^Ga-oxides or ^68^Ga-hydroxides, to complexes with EDTA or trifluoroacetate as ligands.

Another stationary phase was examined using Whatman Grade 1 Chr as the stationary phase and the eluent was a mixture of water:ethanol:pyridine (4:2:1) [23,27]. Here, [^68^Ga]Ga-EDTA produced a clean peak, whereas the ^68^Ga-colloid peak was not sharp (Table 4, entry 5).

The results presented here indicate that iTLC-SG strips do not provide a perfect stationary phase for the development of [^68^Ga]Ga-EDTA chromatograms, since the obtained peaks were wide, asymmetric and tailed. Due to this observation, it was decided to proceed with Whatman Grade 1 Chr strips for further trials. Of all the combinations tested in this study, only one system resulted in ^68^Ga-colloid peaks, with Rf = 0 (Table 4, entry 1). The others showed some transchelation, resulting in Rf > 0.

To address this issue, we also investigated HCl with pH = 5.6 as an eluent, i.e. the same pH as the method used to prepare ^68^Ga-colloid. The simple mobile phase of HCl, adjusted with NaOH to pH = 5.6, gave the best and most well-defined ^68^Ga-colloid peak using both iTLC-SG and Whatman Grade 1 Chr as stationary phases (Table 5, entries 1 and 2). Thus, it was concluded that a chromatography system consisting of the combination of Whatman Grade 1 Chr and HCl (pH 5.6) was the optimum method for quality control of the labelled product (Table 5, entry 2). Additionally, since this method required the adjustment of HCl to pH = 5.6 with NaOH, we also investigated whether the use of saline as a mobile phase instead of HCl was useful. Table 5, entry 3, demonstrates this alternative method of using a system consisting of Whatman Grade 1 Chr as a stationary phase and saline as a mobile phase as a routine quality control for [^68^Ga]Ga-EDTA. The advantage of this method is that it is simple, fast, cheap and reproducible.

In this study, we do not expect the presence of free [^68^Ga]Ga^3+^ in the product, with the hypothesis being that any free ions would either coordinate to EDTA or form ^68^Ga-colloid immediately under the production conditions. To provide supporting evidence for this assumption, we created three solutions, ((i) generator eluate, (ii) kit without EDTA and (iii) [^68^Ga]Ga-EDTA product), on which a) paper chromatography using the optimized paper chromatography method and b) HPLC were performed to determine EDTA content, as described in Section 2.2.2 and Section 2.1.3, respectively.

- Generator eluate consisting of [^68^Ga]GaCl_3_ was analyzed using Whatman paper, showing that radioactivity developed to the eluent front only as [^68^Ga]Ga-EDTA. Therefore, if free [^68^Ga]Ga^3+^ exists, it cannot be separated from the intended product.
- The ^68^Ge/^68^Ga-generator was eluted into a kit prepared without EDTA (analogous to the production of [^68^Ga]Ga-EDTA). On the Whatman paper, the product stayed at Rf = 0 showing that, if EDTA is not present, free [^68^Ga]Ga^3+^ does not exist in the solution.
- The intended [^68^Ga]Ga-EDTA product analyzed using HPLC provided a single clear peak at Rt = 5.9 min. However, the eluate solution and the kit without EDTA did not produce signals on the HPLC chromatograms, thus indicating that ^68^Ga was trapped on the HPLC column in both cases.

These observations confirm that there is no considerable free [^68^Ga]Ga^3+^ present in the product solution and, as such, it is not necessary to analyze this under general production.

## 4. Materials and Methods

### 4.1. EDTA kit Production

The raw materials used to prepare 100 mL of EDTA kit solution were: TRIPLEX III (ethylenedinithrilotetraacetic acid disodium salt dihydrate = Na_2_EDTA·2H_2_O; Merck, 1.37004.1000, VWR), sodium acetate trihydrate (Merck, 1.06235.1000, VWR), sodium hydroxide: sterile 3 M solution 10 × 10 mL (Hospital Pharmacy) and sterile water (SAD, solvent for parenteral use, 100 mL bottles).

EDTA kit production was aseptically performed in a Laminar Air Flow cabinet (GMP grade A) situated in a clean room (grade C), with microbiological monitoring using settle plates and particle monitoring (MET One 3415 particle counter) under the complete duration of critical processing.

In the clean room, reagents were weighed in sterile weight bottles with their lids and transferred to the LAF-cabinet. In the LAF-cabinet, reagents were transferred to the volumetric flask (100 mL) and dissolved in sterile water. After complete dissolution using a magnet stirrer, the product was sterile-filtered (filter unit Cathivex GV 0.22 μm Merck Millipore), portioned manually with a Finnpipette with a sterile tip and sealed in sterile 10 mL glass vials with 3 mL of product in each vial. Vials were frozen at −18 °C.

The ingredients for 100 mL EDTA kit solution are shown in Table 6.

### 4.2. EDTA Kit Quality Control

The sterile filter used for the bulk production was tested manually for integrity. For this purpose, 3 mL of sterile water was drawn into a 10 mL syringe, followed by air. The filter was then attached to the syringe and the syringe’s content rapidly expelled. The filter was intact if the syringe’s piston returned to its starting position. The produced EDTA kits were individually tested visually for appearance and volume, determined by comparison to a standard volume. The product pH was measured on a single sample using a calibrated ISO 9001 certified pH-meter (HACH HQ411d with provided PHC705 electrode). Sterility testing of a single sample of the product was carried out by the hospital microbiology department to determine the amount of CFU in the product.

The content of Disodium EDTA dihydrate in the EDTA kit was measured using High-Pressure Liquid Chromatography analysis (HPLC), based on a complexation reaction between EDTA and Fe^3+^ (formation constant for Fe-EDTA, K_f_ = 1.3 × 10^25^) [14], reversed phase column and ion pair reagent, as described in [14,15,16]. The HPLC system consisted of LC-20AD UFLC Shimadzu pump, SPD-20A HPLC UV-VIS detector (wavelength 254 nm), chromatographic column (Kinetex 5 μm XB-C18 100a 150 × 4.6 mm) and associated LabSolutions software. The following HPLC parameters were used: flow: 1 mL/min; injection volume: 30 μL; eluent: 4.5 g sodium acetate trihydrate with 800 mL of water added, pH adjusted to 4.0. Thereafter, 4.0 g tetrabutylammonium bromide was added and filled up to 1 L with water. To create a calibration curve, 10 reference samples of Na_2_EDTA dihydrate in water were produced with concentrations ranging from 0.01 mg/mL to 0.1 mg/mL in steps of 0.01 mg/mL. A solution of FeCl_3_·6H_2_O in 30% acetic acid/water (*V/V*) with a concentration of 0.175 mg/mL was produced. For HPLC analysis, 10 samples were prepared by mixing the Na_2_EDTA dihydrate reference sample and FeCl_3_·6H_2_O solution 1/1. Samples from EDTA kits were diluted 1/10 with water and mixed with FeCl_3_·6H_2_O solution 1/1 prior to HPLC analysis.

Finally, a complete labelling reaction of an EDTA kit with ^68^Ga, producing [^68^Ga]Ga-EDTA according to the procedure described below, was required for approval of each EDTA kit batch.

### 4.3. [^68^Ga]Ga-EDTA Production

The production of [^68^Ga]Ga-EDTA was performed in a grade C clean room, with all critical processes conducted in a GMP-grade A Laminar Air Flow cabinet. Microbiological monitoring with settle plates, glove print and particle monitoring (MET One 3415 particle counter) was performed for the complete duration of critical processing. Synthesis was performed automatically using a PharmTracer ModularLab. 

The critical sterile procedure in which the thawed, sterile, sealed vial with 3 mL of on-site, pre-produced EDTA kit, was equipped with a vent needle and a needle with a filter unit for sterile filtering (Cathivex GV 0.22 mm Merck Millipore), which was performed in a GMP-grade A LAF cabinet. The product vial was then connected to the elution cassette’s outlet and placed into the shielded container, after which PharmTracer’s elution software was executed.

**Figure 6:**
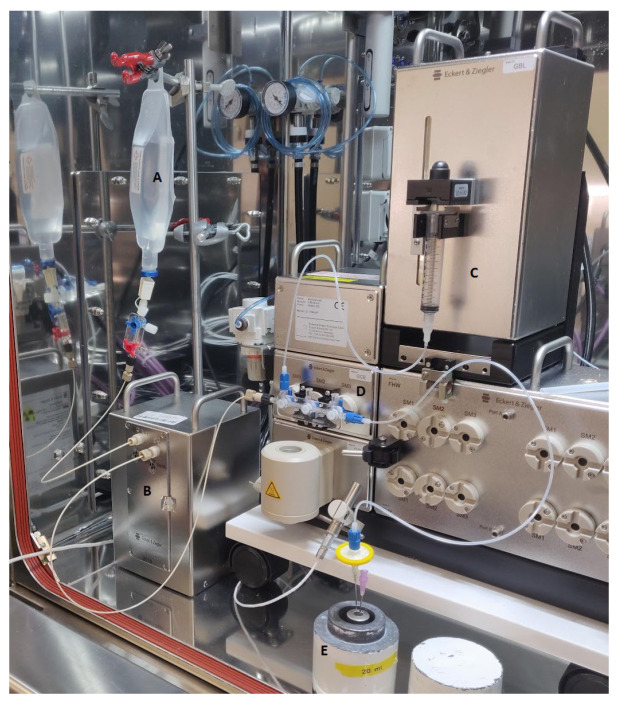
Synthesis setup. A: 0.1 N HCl; B: GalliaPharm ^68^Ge/^68^Ga-generator; C: dispensing syringe; D: Stopcock manifold; E: lead shield with product vial.

The GMP-compliant (production and test compliance with Ph.Eur. and DMA regulations) GalliaPharm ^68^Ge/^68^Ga-generator (Eckert and Ziegler 1.85 GBq), was eluted with 7 mL of sterile ultrapure 0.1 N HCl for the direct elution of GalliaPharm into the thawed sterile, sealed vial containing the on-site pre-produced EDTA kit. The process was fully automated using the elution cassette for Modular-Lab PharmTracer (Eckert and Ziegler Eurotop GmbH), Figure 6.

The use of Modular-Lab PharmTracer system, together with Software Modular-Lab and elution/synthesis cassettes (Eckert and Ziegler Eurotope GmbH), provides an efficient, routine GMP production of radiopharmaceuticals and prevents cross-contamination issues. The system was fully qualified and validated to perform [^68^Ga]Ga-EDTA synthesis based on the use of 0.1 N HCl for the elution of the ^68^Ge/^68^Ga-generator. Additionally, the automatic synthesis was reproducible and provided the benefit of reductions in the radiation dose to the staff. The elution cassette was inserted into the PharmTracer’s stopcock manifold, and the generator connected to the inlet port of the cassette. 

The shielded GalliaPharm system is an approved radionuclide generator for medical use, allowing for the elution of [^68^Ga]GaCl_3_ from a titanium dioxide column, onto which the parent radionuclide ^68^Ge is adsorbed. ^68^Ga is eluted using sterile ultrapure 0.1 N HCl. The sterile ultrapure HCl solution was connected to the generator’s inlet port and the eluate collected at the outlet port with intended use in medicinal production. The generator was eluted a minimum of 24 h prior to labeling in order to avoid the accumulation of free long-life ^68^Ge ions and metal impurities, e.g., zinc ions (Zn^2+^) arising as the decay product from ^68^Ga, which can interfere with the labeling reaction [2,28].

The ^68^Ga eluate was regularly investigated for sterility as well as for ^68^Ge breakthrough by a gamma spectrum test in the laboratory. Other potential metal ion impurities, such as Fe and Zn ions, were defined by the manufacturer (Eckert and Ziegler, Berlin, Germany) as being lower than the levels allowed by the European Pharmacopeia.

### 4.4. [^68^Ga]Ga-EDTA Quality Control

Routine quality control of the labelled product included pH verification with indicator papers and the visual determination of appearance and volume, as assessed by comparison with a standard. Testing of the filter integrity was performed as described earlier for EDTA kits and radionuclide purity was tested by measuring the half-life and ^68^Ge-breakthrough in a product sample. The product's radionuclide identity was confirmed by half-life determination according to the Ph.Eur. monograph for ^68^Ge/^68^Ga generators: three measurements of radioactivity within 15 min in a dose calibrator (Capintec CRC-55TR), which was routinely checked for stability and accuracy. Results were plotted logarithmically as a function of time. ^68^Ge breakthrough was determined at a minimum 48 h after the [^68^Ga]Ga-EDTA end of synthesis (EOS) in an automatic gamma-counter (Perkin Elmer, Wizard 2480). In accordance with the Ph.Eur., the results were expressed as a percentage of total eluted ^68^Ga. 

Standard paper chromatography method was used to determine the radiochemical purity (RCP) and identity of [^68^Ga]Ga-EDTA. The product was applied 2 cm from the bottom of a Whatman Grade 1 Chr (GE Healthcare) chromatography paper strip (12 cm × 2 cm) and directly transferred to a chromatography tank with 10 mL of 0.9% sodium chloride (NaCl). When the solvent front reached 8 cm from the bottom, the strip was removed from the tank and analyzed using a LabLogic TLC-scanner Scan-RAM (Laura software and PS Plastic/PMT radio-detector; 120 mm; speed 1 mm/s). To validate this paper chromatography method, 0.9% NaCl was used as an eluent and Whatman Grade 1 Chr plates as the stationary phase; a standard solution of [^68^Ga]Ga-EDTA (product) and a reference solution of ^68^Ga-colloid (impurities) were used. Since the presence of ^68^Ga-ions was not expected in a product with large excess of EDTA, ^68^Ga-ions were not considered. [^68^Ga]Ga-EDTA was produced using the method described in the previous method section. ^68^Ga-colloid was prepared using the bench titration of ^68^Ga-eluate with sodium hydroxide solutions to pH 5.6 ± 0.2 [22].

Quantitative endotoxin detection in the product was measured by an EndoSafe Nexgen PTS (Charles River) spectrophotometer, which utilized disposable cartridges with Limulus amebocyte lysate (LAL) reagents (product number PTS201F).

Additional extended quality control procedures (XQC), carried out at regular intervals, included: (1) gamma spectrum measurement by a high purity germanium detector; (2) sterility testing analyzed by the Department of Microbiology, as described above, for EDTA kits and (3) stability studies (repetition of pH and RCP/ID measurements), no less than 2 h post-EOS. 

## 5. Conclusions

This paper describes the development and validation of methods for the production and quality control of a simple EDTA kit and its labeling with ^68^Ga. Both production procedures are performed under aseptic conditions compliant with GMP regulations. 

Quality control of both the simple kit and labelled product consisted of a visual assessment, volume designation, pH measuring, filter integrity test and test for sterility. The EDTA kit was controlled using the HPLC method to measure precursor (EDTA) content. The final product, [^68^Ga]Ga-EDTA, was controlled for radiochemical purity using the developed, validated and established paper chromatography method with Whatman Grade 1 Chr strips as the solid phase and 0.9% NaCl as an eluent, together with measurements of the radionuclidic purity from the determination of the half-life, gamma spectrum and ^68^Ge-breakthrough.

It was shown that the developed processes are reliable, highly reproducible, and easily implemented for local clinical use.

## Figures and Tables

**Table 1:** Composition of an EDTA kit.

Reagent	Amount per EDTA Kit
Disodium EDTA dihydrate	1.86 mg
Sodium acetate trihydrate	136 mg
NaOH	7.99 mg
Sterile water	Up to 3.00 mL

**Table 2:** Specifications and validation of three productions of EDTA kits.

Test (Method) *	Specifications	EDTA Kit Batch 1	EDTA Kit Batch 2	EDTA Kit Batch 3
pH (pH meter)	12.0–13.0	12.4	12.3	12.4
Filter integrity (Manual)	Intact	Intact	Intact	Intact
Sterility	No growth	No growth	No growth	No growth
Volume (Visual)	3.0 ± 0.5 mL	3.0	3.0	3.0
Appearance (Visual)	Clear without particles	Clear without particles	Clear without particles	Clear without particles
Identity (Fe-EDTA) (HPLC)	2.5–3.5 min	3.0	3.0	3.0
Na_2_EDTA·2H_2_O (HPLC)	0.62 g/mL ± 20% (0.50–0.74 mg/mL)	0.64	0.69	0.69
Labelling (full QC program)	Comply	Comply	Comply	Comply

* Results are from analyses performed immediately after production, except the HPLC results, which are obtained after 14 months.

**Table 3:** Specifications and validation for three productions of [^68^Ga]Ga-EDTA.

QC (Method) *	Specifications	[^68^Ga]Ga-EDTA Batch 1	[^68^Ga]Ga-EDTA Batch 2	[^68^Ga]Ga-EDTA Batch 3
Radioactivity (dose calibrator)	≤1373 MBq at EOS	1363	1373	1329
Volume (visual)	9.0–11.0 mL	9.0	9.5	9.5
Appearance (visual)	Clear without particles	Clear without particles	Clear without particles	Clear without particles
Filter integrity (manual)	Intact	Intact	Intact	Intact
pH (indicator paper)	4.0-6.0	4.7	4.7	4.7
^68^Ga-colloid (paper chromatography)	<3%	0.3	0.3	0.2
RCP (paper chromatography)	>95%	99.8	99.7	99.8
Identity (paper chromatography)	0.7 < Rf < 1.3	1.1	1.0	1.1
Radionuclidic purity (Gamma counter)	<0.001% activity from ^68^Ge	<0.00001	<0.00001	<0.00001
Endotoxin (EndoSafe)	<17.5 EU/mL	<5.00	<5.00	<5.00
Sterility	No growth	No growth	No growth	No growth

* Displayed results are from analyses performed immediately after production.

**Table 4:** Results from TLC-studies with reference to the literature. SP: stationary phase, MP: mobile phase.

Entry	TLC System	^68^Ga-Colloid	[^68^Ga]Ga-EDTA
1	SP: iTLC-SG MP: 77 g/L NH_4_OAc_(aq)_/MeOH (1/1) ^68^Ga-colloid: Ph.Eur. Reference: [21]	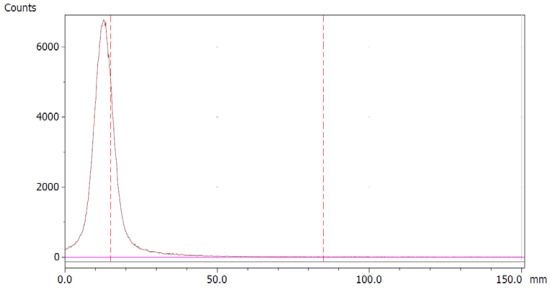	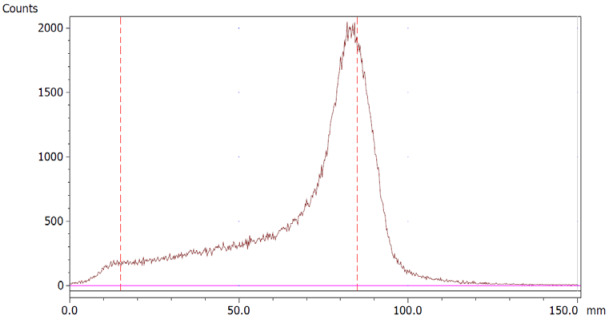
2	SP: iTLC-SG MP: 0.1M EDTA in 0.25 M NH_4_Ac, pH 5.5 ^68^Ga-colloid: Ph.Eur. Reference: [24]	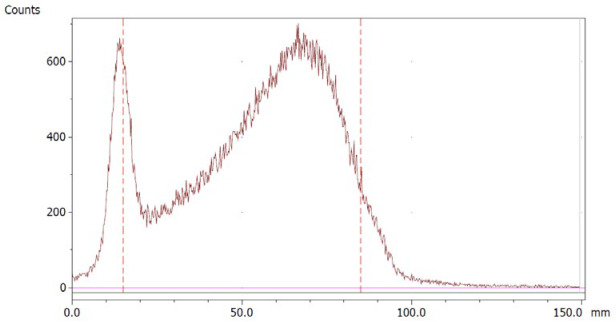	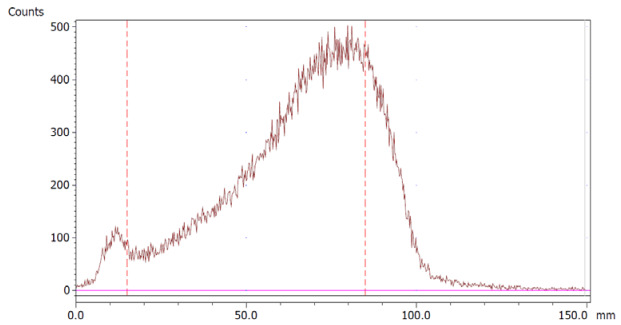
3	SP: iTLC-SG MP: TFA 4% ^68^Ga-colloid: Ph.Eur. Reference: [25]	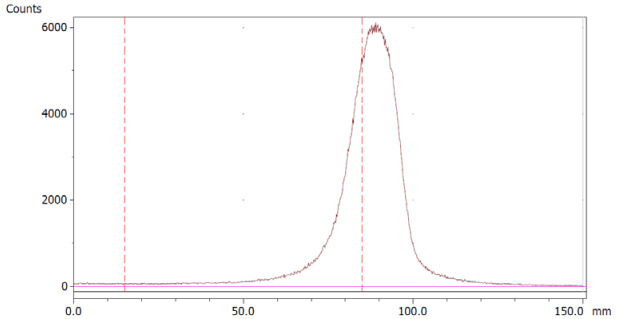	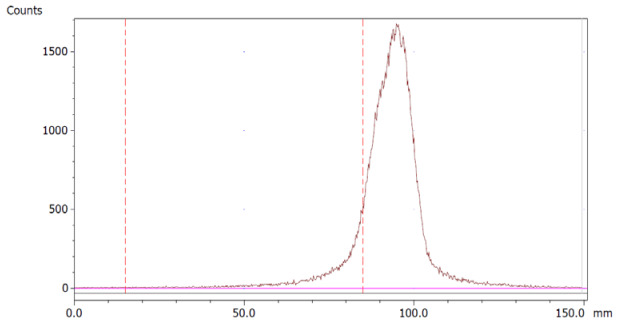
4	SP: iTLC-SG MP: 0.9% NaCl/MeCN (1/1) + 0.08% TFA ^68^Ga-colloid: Bench titration Reference: [26]	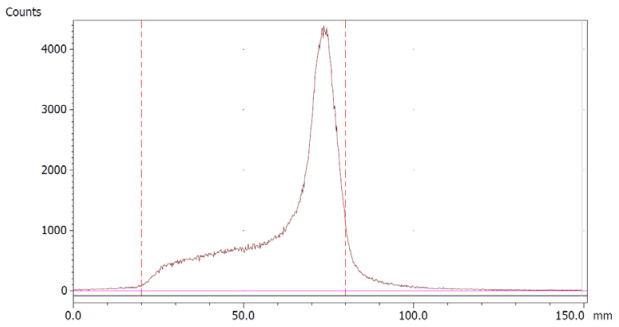	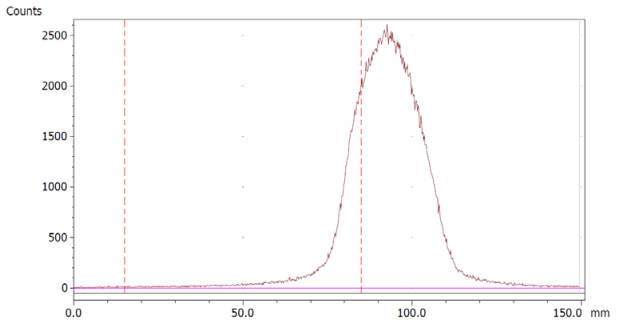
5	SP: Whatman Grade 1CHR MP: Water/ethanol/pyridine (4/2/1) ^68^Ga-colloid: Ph.Eur. Reference: [23,27]	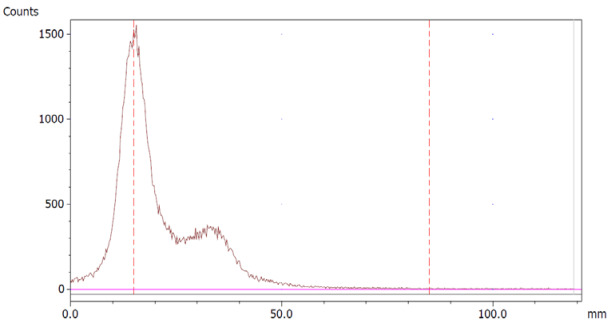	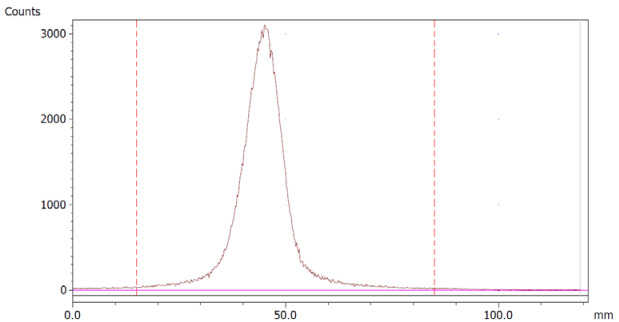

**Table 5:** Results from TLC- and paper chromatograpy studies using local combinations of stationary and mobile phases with inspiration taken from the results presented in Table 4. SP: stationary phase, MP: mobile phase.

Entry	TLC System	^68^Ga-Colloid	[^68^Ga]Ga-EDTA
1	SP: iTLC-SG MP: HCl pH 5.6 ^68^Ga-colloid: Bench titration	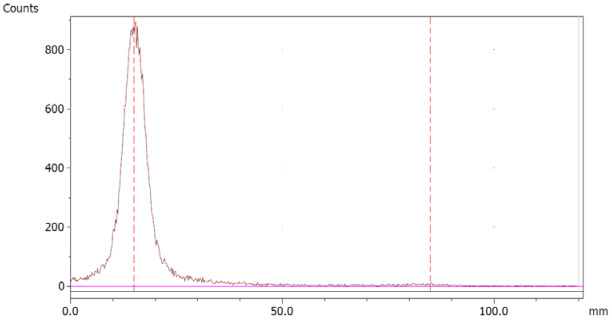	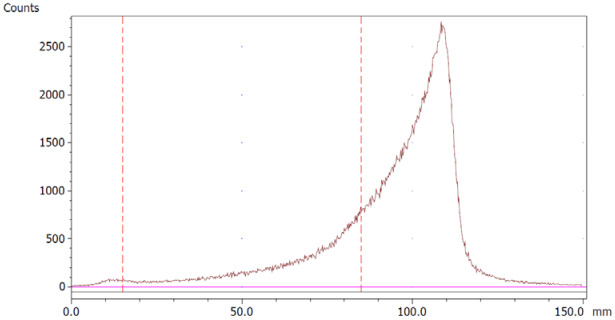
2	SP: Whatman Grade 1CHR MP: HCl pH 5.6 ^68^Ga-colloid: Bench titration	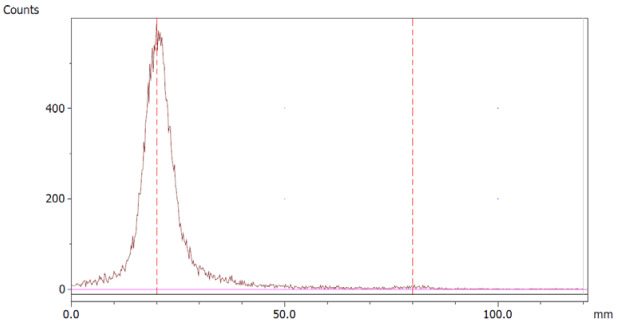	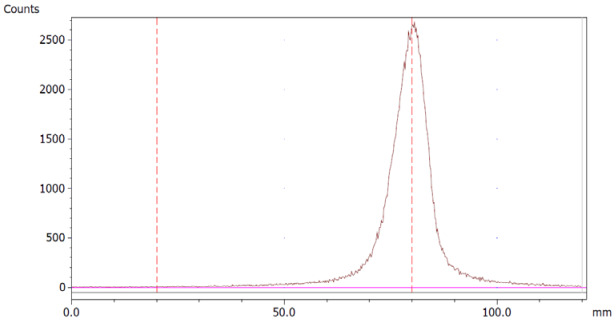
3	SP: Whatman Grade 1CHR MP: 0.9% NaCl ^68^Ga-colloid: Bench titration	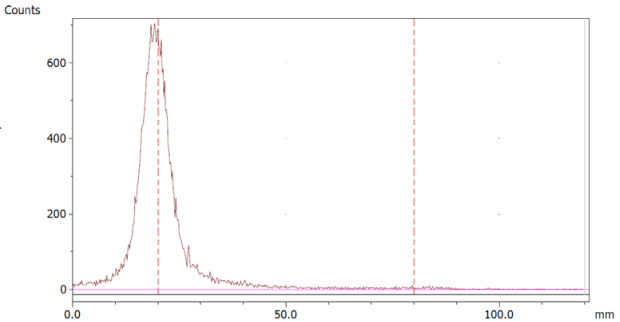	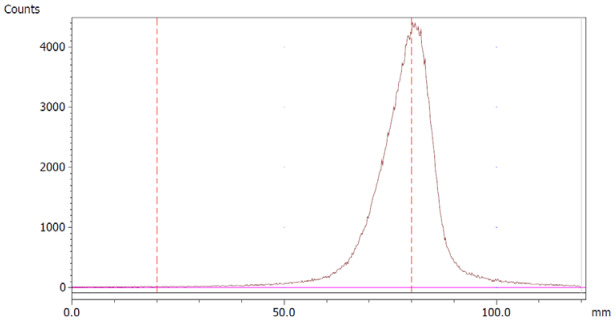

**Table 6:** Amount of reagents in 100 mL EDTA kit solution.

Reagent	Molar Weight (g/mol)	Amount of Substance (mol)	Mass (g)	Volume (mL)
Disodium EDTA dihydrate	372.24	0.000167	0.0620	-
Sodium acetate trihydrate	136.08	0.0333	4.54	-
NaOH (3 M)	-	0.00666	-	2.22
Sterile water	-	-	-	Up to 100

## Data Availability

The data presented in this study are available on request from the corresponding author.
